# GRACE Score among Six Risk Scoring Systems (CADILLAC, PAMI, TIMI, Dynamic TIMI, Zwolle) Demonstrated the Best Predictive Value for Prediction of Long-Term Mortality in Patients with ST-Elevation Myocardial Infarction

**DOI:** 10.1371/journal.pone.0123215

**Published:** 2015-04-20

**Authors:** Simona Littnerova, Petr Kala, Jiri Jarkovsky, Lenka Kubkova, Krystyna Prymusova, Petr Kubena, Martin Tesak, Ondrej Toman, Martin Poloczek, Jindrich Spinar, Ladislav Dusek, Jiri Parenica

**Affiliations:** 1 Institute of Biostatistics and Analyses, Masaryk University, Brno, Czech Republic; 2 Department of Cardiology, University Hospital Brno, Brno, Czech Republic; 3 Faculty of Medicine, Masaryk University, Brno, Czech Republic; 4 International Clinical Research Center–Department of Cardiovascular Disease, St Anne’s University Hospital, Brno, Czech Republic; 5 Hospital Podlesi A.S., Trinec, Czech Republic; 6 Hospital Trebic, Trebic, Czech Republic; Scuola Superiore Sant'Anna, ITALY

## Abstract

**Aim:**

To compare the prognostic accuracy of six scoring models for up to three-year mortality and rates of hospitalisation due to acute decompensated heart failure (ADHF) in STEMI patients.

**Methods and Results:**

A total of 593 patients treated with primary PCI were evaluated. Prospective follow-up of patients was ≥3 years. Thirty-day, one-year, two-year, and three-year mortality rates were 4.0%, 7.3%, 8.9%, and 10.6%, respectively. Six risk scores—the TIMI score and derived dynamic TIMI, CADILLAC, PAMI, Zwolle, and GRACE—showed a high predictive accuracy for six- and 12-month mortality with area under the receiver operating characteristic curve (AUC) values of 0.73–0.85. The best predictive values for long-term mortality were obtained by GRACE. The next best-performing scores were CADILLAC, Zwolle, and Dynamic TIMI. All risk scores had a lower prediction accuracy for repeat hospitalisation due to ADHF, except Zwolle with the discriminatory capacity for hospitalisation up to two years (AUC, 0.80–0.83).

**Conclusions:**

All tested models showed a high predictive value for the estimation of one-year mortality, but GRACE appears to be the most suitable for the prediction for a longer follow-up period. The tested models exhibited an ability to predict the risk of ADHF, especially the Zwolle model.

## Introduction

Mortality of individuals with ST-segment elevation myocardial infarction (STEMI) is influenced by many factors. Of these factors, age, heart failure, time delay to treatment, mode of treatment, history of prior myocardial infarction (MI), diabetes mellitus, renal failure, number of diseased coronary arteries, ejection fraction (EF), in-hospital events (e.g., bleeding, cardiac arrest, progression of heart failure) are important [1–6]. The in-hospital mortality of STEMI patients treated by primary percutaneous coronary intervention (pPCI) varies between 2.7% and 8.0% [7] and six-month mortality is about 12%, with higher mortality rates among high-risk patients [6].

Several risk scores have been developed in order to stratify STEMI patients according to their high/low risk of mortality or complications. The Thrombolysis in Myocardial Infarction (TIMI) risk score was originally focused on the 30-day mortality [8]. Subsequently, it was validated for STEMI patients treated by pPCI and for prediction of one-year mortality [9,10]. The Zwolle score was created for the prediction of 30-day mortality to identify low-risk patients suitable for an early discharge from hospital [4]. The Primary Angioplasty in Myocardial Infarction (PAMI) risk score is used to predict the six-month mortality. The Controlled Abciximab and Device Investigation to Lower Late Angioplasty Complications (CADILLAC) risk score is used to predict the one-year mortality. The development of both risk scores (PAMI and CADILLAC) was based on individuals treated by invasive procedures [2]. The Global Registry of Acute Cardiac Events (GRACE) risk score was calculated for six-month mortality based on a robust registry cohort for the entire spectrum of acute coronary syndromes, and was subsequently refined and simplified [1,11]. The dynamic TIMI risk model is an upgrade of the classic TIMI risk score, using in-hospital events for an easy reassessment of the risk of patients discharged from hospital [3] (Table 1).

**Table 1:** Risk-scoring models and their components.

	TIMIMorrow [8], 2000	Zwolle De Luca [3], 2004	PAMI Addala[1], 2004	CADILLAC Halkin [5], 2005	GRACE Fox [11], 2006	Dynamics TIMI Morrow [2], 2013
**Time of prediction**	**1 year**	**30 days**	**6 months**	**1 year**	**6 months**	**1 year**
**c-statistic—AUC**	**0.73**	**0.91**	**0.77**	**0.82**	**0.85**	**0.81**
Age	**x**	**x**	**x**	**x**	**x**	**x**
Diabetes mellitus	**x**		**x**			
Hypertension	**x**					
Angina pectoris	**x**					
Low systolic blood pressure	**x**				**x**	**x**
Heart rate	**x**		**x**		**x**	**x**
Heart failure	**x**	**x**	**x**	**x**	**x**	**x**
Weight	**x**					**x**
Anterior MI or LBBB	**x**	**x**	**x**			**x**
Ischaemia time	**x**	**x**				**x**
Cardiac arrest					**x**	
ST segment deviation					**x**	
Elevated cardiac enzymes					**x**	
Creatinine/renal insufficiency				**x**	**x**	
TIMI flow after PCI		**x**		**x**		
Three-vessel disease		**x**		**x**		
Ejection fraction				**x**		
Anaemia				**x**		
Hospital event*						**x**

*Recurrent MI, stroke, major bleed, CHF/shock, arrhythmia, renal failure.

BP—blood pressure, LBBB—left bundle branch block; PCI—percutaneous coronary intervention.

As mentioned above, several factors might influence the predictive accuracy of risk scores in STEMI patients. These risk scores are used for the prediction of short-term survival/complications, but none of them have been tested for the follow-up of a longer period. Therefore, application of these risk scores for long-term follow-up might provide very interesting findings.

The aim of our study was to compare six scoring models for the prediction of up to three-year mortality and, at the same time, to ascertain whether these models are useful for the estimation of risk of rehospitalisation due to acute decompensated heart failure (ADHF) in a cohort of consecutive STEMI patients treated by pPCI.

## Methods

The study protocol complied with the Declaration of Helsinki and was approved by the Ethics Committee of the University Hospital Brno (Brno, Czech Republic) and by the Ethics Committee of the Masaryk University (Brno, Czech Republic). Written informed consents were obtained from all subjects before their participation in the study.

### Study population

From November 2005 to October 2008, 913 patients with STEMI were referred for primary percutaneous coronary intervention (PCI). They were admitted to the Coronary Care Unit (CCU) of the Department of Cardiology at University Hospital Brno (Table 2). The exclusion criteria involved: age >80 years (n = 112); known or recently diagnosed cancer; inflammatory or connective-tissue disorders; disease other than cardiovascular disease that could clearly limit the one-year prognosis (n = 48); refusal to sign the informed consent or non-compliance (n = 74); remoteness (distance from the place of residence to the hospital larger than 100 km, ruling out the possibility of follow-up; n = 86). The study cohort included a total of 593 patients. All patients were of Caucasian origin. In accordance with the current ESC guidelines [12], dual antiplatelet therapy, angiotensin-converting enzyme inhibitors, beta-blockers and statins were employed as the standard recommended treatment.

**Table 2:** Baseline characteristics of patients and medical therapy upon hospital admission.

N = 593	N (%)/median (5^th^; 95^th^ percentile)
Sex (female)	149 (25.1%)
Age (years)	63 (45; 79)
Height (cm)	174 (159; 185)
Weight (kg)	82 (63; 109)
Body mass index (kg/m^2^)	28.0 (22.0; 35.0)
Systolic blood pressure (mmHg)	140 (90; 185)
Diastolic blood pressure (mmHg)	80 (55; 105)
Heart rate (min^–1^)	73 (52; 107)
Smoker	254 (42.8%)
Previous unstable angina pectoris	205 (34.6%)
Hypertension	335 (56.5%)
Diabetes mellitus	160 (27.0%)
Hyperlipoproteinaemia	493 (83.1%)
History of myocardial infarction	72 (12.1%)
History of PCI/CABG	39 (6.6%)
History of stroke or TIA	30 (5.1%)
Chronic obstructive pulmonary disease	19 (3.2%)
Atrial fibrillation	13 (2.2%)
Chronic angina pectoris	84 (14.1%)
Anti-aggregation (upon admission)	119 (20.1%)
ACEI and/or ARB (upon admission)	184 (31.0%)
Beta-blockers (upon admission)	158 (26.6%)
Statins (upon admission)	98 (16.5%)
*Adverse event during hospitalisation	23 (3.9%)
AHF mild (Killip II)	127 (21.4%)
Pulmonary oedema (Killip III)	14 (2.4%)
Cardiogenic shock (Killip IV)	22 (3.7%)
Time to treatment (min)	238 (124; 660)

*Recurrent MI, stroke, major bleed, CHF/shock, arrhythmia, renal failure;

MI—myocardial infarction, ACEI—angiotensin-converting enzyme inhibitor, ARB—angiotensin II receptor blockers, PCI—percutaneous coronary intervention, CABG—coronary artery bypass grafting, TIA—transient ischaemic attack, AHF—acute heart failure.

The diagnosis of STEMI was based on MI symptoms, appropriate electrocardiographic changes (ST-segment elevation at the J point in two contiguous leads with a cutoff value of ≥0.1 mV in all leads (including V7-9), in leads V2–V3 with a cutoff ≥0.2 mV in men aged ≥40 years and ≥0.25 mV in men <40 years or ≥0.15 mV in women) or with a new left branch bundle block, and elevation of troponin I. Time from the onset of chest pain to pPCI was <12 h. The diagnosis of acute heart failure (AHF) was made according to clinical signs upon hospital admission and then during hospitalisation based on the Killip classification.

### Laboratory methods

Blood samples for standard biochemical and haematological blood tests were taken immediately upon hospital admission before pPCI and 24 h after the onset of chest pain, and included the troponin-I ADV assay (Abbott Laboratories, Abbott Park, IL, USA).

### Echocardiography

Echocardiographic assessment was carried out 3–5 days after MI onset. Left ventricular ejection fraction was estimated primarily using the bi-plane Simpson’s formula with apical two- and four-chamber views (90% of values). The remaining values were obtained using the Teichholz formula. Echocardiography was assessed using a Vivid 7 device (GE Vingmed Ultrasound, Horten, Norway).

### Invasive measurement

A diseased coronary vessel was defined as having at least one reduction of intraluminal diameter >50% on major coronary arteries (left main, left anterior descending, left circumflex, or right coronary artery) or their branches with diameter ≥1.5 mm. Significant left main artery stenosis was coded as “two-vessel disease”. TIMI flow grade was assessed before and after pPCI.

### Follow-up

Patients were monitored prospectively as outpatients in the cardiology department of our hospital. Surviving patients were followed up for at least 3 years.

### Endpoints

The primary endpoint was all-cause mortality; the secondary endpoint was hospitalisation due to AHF, as authors consider it to be an endpoint with a strong relationship to previous myocardial infarction and, at the same time, the one which most affects the quality of life. All-cause mortality was known for all patients, but not the exact number of cardiac deaths. According to data from the central registry of the Czech Republic, there were no traumatic deaths, Myocardial re-infarction, stroke and unplanned coronary revascularisation (PCI or coronary artery by-pass grafing) were also recorded. All endpoints were determined by two cardiologists, who based their judgment on discharge reports.

### Statistical analyses

Data analysis was carried out using SPSS v21.0.0 (IBM, Armonk, NY, USA). Discrete variables are presented as frequency counts and percentages. Continuous variables are expressed as the median (5^th^ and 95^th^ percentile range).

The discriminative ability of risk scores was evaluated by the area under the receiver-operating characteristics (ROC) curve (AUC). The endpoints of interest were mortality and rehospitalisation each week from 1 week to 152 weeks (0–3 years), focusing especially on mortality and rehospitalisation at one, two and three years. The statistical significance of differences between AUCs was tested by the DeLong test.

To depict all-cause mortality at a given time point (1, 6, 12, 24, 36 months), a landmark survival analysis based on the Kaplan–Meier estimate was used. The level of significance was set at α = 0.05.

## Results

Baseline characteristics of 593 patients and medications used before the hospital admission are listed in Table 2. Each individual was followed up for at least three years (the median follow-up was 50.4 months).

Selective coronarography revealed three-vessel disease in 158 (26.6%) patients; 344 (58.0%) patients had TIMI flow of zero upon hospital admission and a value of 8 (1.3%) after pPCI (Table 3). The remaining laboratory and invasive parameters are listed in Table 3. Treatment modalities administered during hospitalisation and upon hospital discharge are shown in Table 4.

**Table 3:** Characteristics of laboratory tests and invasive procedures.

N = 593	N (%)/median (5^th^; 95^th^ percentile)
Number of diseased vessels	
1	227 (38.3%)
2	208 (35.1%)
3	158 (26.6%)
IRA	
Left main, LAD or CABG—LAD	(43.7%)
RCA, RPLD, RIVP, CABG-RCA	(36.8%)
RCx, RMS, RIM, CABG-RCx,-RMS	82 (8.3%)
RD	14 (2.4%)
IRA—collaterals	148 (25.0%)
Initial TIMI flow 0	344 (58.0%)
Final TIMI flow 0	8 (1.3%)
Creatinine (upon admission) (μmol/l)	88 (61; 137)
Glycemia (upon admission) (mmol/L)	8.0 (6.0; 17.0)
Hemoglobin (upon admission) (g/L)	145 (121; 166)
Troponin I (max) (pg/mL)	50 (3; 183)
BNP (upon admission) (pg/mL)	64 (15; 489)
LVEF (echocardiography) (%)	49 (29; 39)

IRA—infarct-related artery, LAD—left anterior descending artery, CABG—coronary artery bypass graft, RCA—right coronary artery, RPLD—ramus posterolateral dexter, RIVP—ramus interventricularis posterior, RCx—ramus circumflexus, RMS—ramus marginalis sinister, RIM—ramus intermedius, RD—ramus diagonalis, BNP—brain natriuretic peptide, LVEF—left ventricular ejection fraction.

**Table 4:** Treatment during hospitalisation and upon hospital discharge.

N = 593	N (%)/median (5^th^; 95^th^ percentile)
PCI of IRA	593 (100%)
Elective PCI of non-IRA during hospitalisation	80 (13.5%)
Acute CABG during initial hospitalisation	2 (0.3%)
ACEI and/or ARB (upon discharge)	545 (91.9%)
Beta-blockers (upon discharge)	555 (93.6%)
Statins (upon discharge)	554 (93.4%)
Diuretics (upon discharge)	167 (28.2%)
Spironolactone (upon discharge)	70 (11.8%)
Duration of hospitalisation (days)	6 (3; 13)

PCI—percutaneous coronary intervention, CABG—coronary artery bypass grafting, ACEI—angiotensin-converting enzyme inhibitor, ARB—angiotensin II receptor blocker

**Fig 1:**
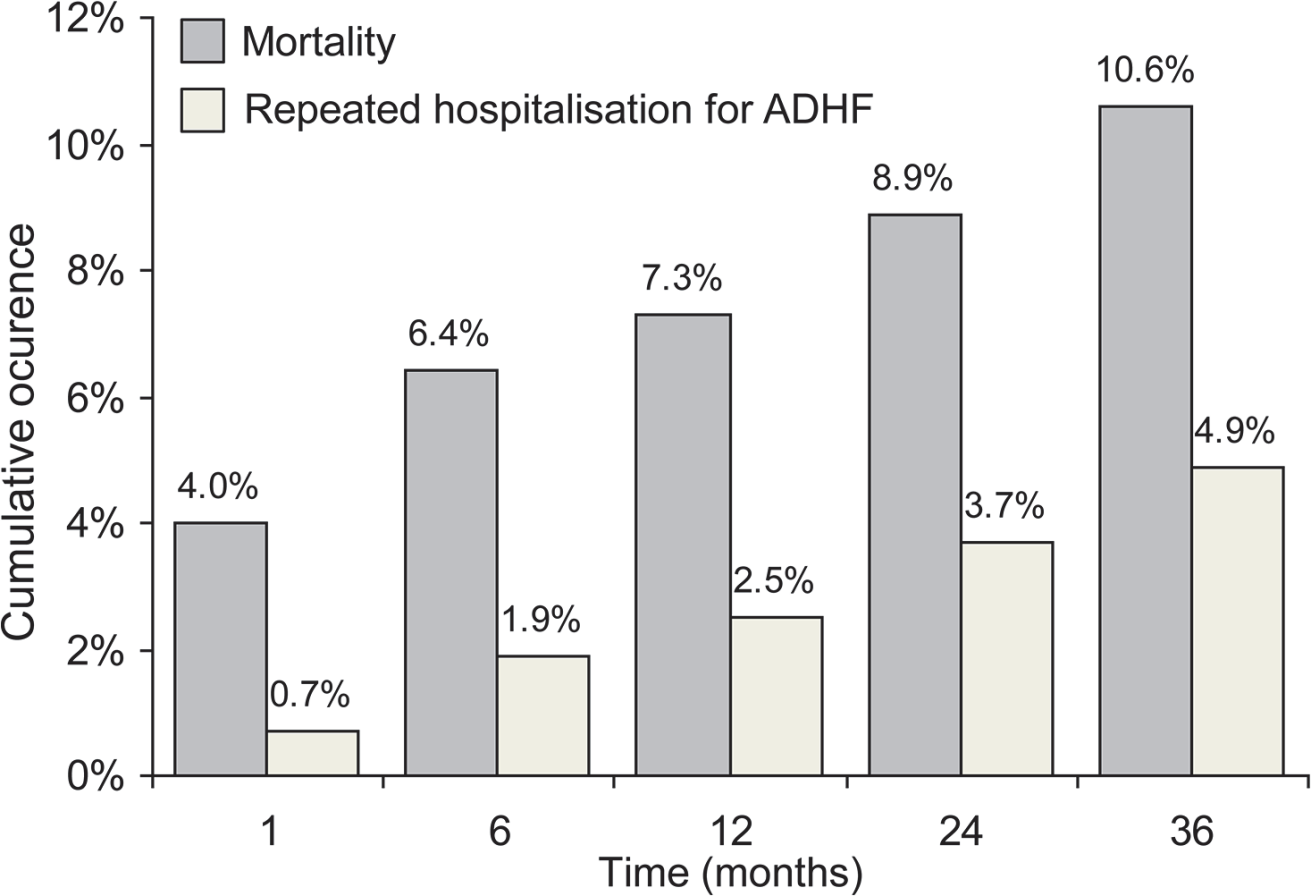
Cumulative occurrence of mortality and repeat hospitalisation for ADHF over time.

pPCI was conducted in 593 patients, and 80 patients (13.5%) underwent elective PCI of a non-culprit coronary lesion during the index hospitalisation (Table 4). In total, 27.7% of patients had the signs and symptoms of AHF upon hospital admission. Thirty-day, one-year, two-year, and three-year mortality rates were 4.0%, 7.3%, 8.9%, and 10.6%, respectively. Repeat hospitalisation due to AHF regardless of mortality was recorded in 0.7%, 2.5%, 3.7%, and 4.9% of patients, respectively (Fig 1). One-, two-, and three-year occurrence of myocardial re-infarction was 4.8%, 6.2% and 7.6%, the respective rates for stroke were 0.8%, 1.9% and 2.2%, and the respective rates for unplanned revascularisation were 7.1%, 9.8% and 10.6%.

**Fig 2:**
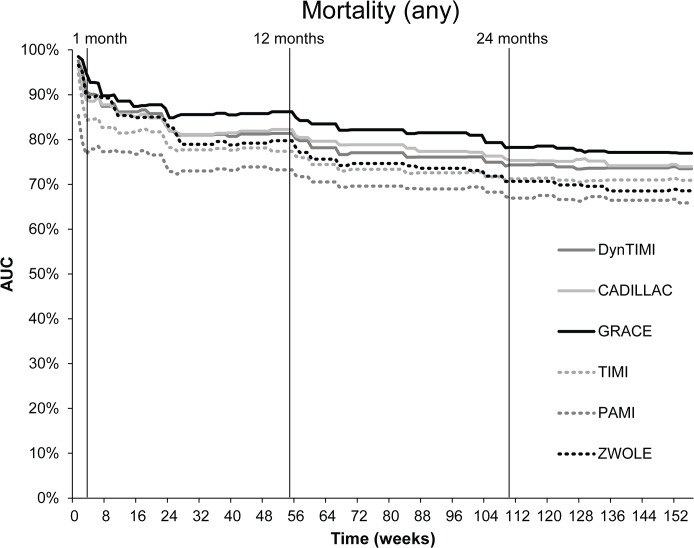
Predictive accuracy of scoring models for mortality during a three-year follow-up period.

The AUC calculated for the six risk models for mortality at each week during the three-year follow-up period is shown in Fig 2. Numerical values of the AUC for mortality and repeat hospitalisation at 6, 12, 24, and 36 months are presented in Table 5. The GRACE risk score had the best predictive accuracy, with an AUC of 0.85 (p<0.001) for six-month mortality and 0.86 (p<0.001) for one-year mortality (Table 5). As for mortality at two years and three years, the AUC of GRACE decreased, but still represented the best predictive tool for long-term mortality: AUC was 0.79 (p<0.001) for 2 years, and 0.77 (p<0.001) for 3 years (Table 5). As for the other risk scores, CADILLAC, dynamic TIMI, and Zwolle also showed a very high predictive accuracy for 6-month and 12-month mortality, with values of AUC ranging between 0.80 and 0.83 (all p<0.001) (Table 5). TIMI and PAMI risk scores were not as good predictors as the other scores, with AUC for 6-month and 12-month mortality ranging between 0.72 and 0.77 (all p<0.001) (Table 5). As regards two- and three-year mortality, the CADILLAC risk score had the next highest predictive accuracy after GRACE: AUC was 0.76 and 0.74, respectively (p<0.001). The worst predictive tools for two- and three-year mortality were TIMI and Zwolle risk scores (Table 5). We compared the predictive values of all six scoring systems. We demonstrated that the GRACE score (which was used as a reference model) was significantly different when compared to PAMI and TIMI scores, and was comparable to CADILLAC, Dynamic TIMI and Zwolle scores (only for 6 and 12 months) (Table 5).

**Table 5:** The AUC of six scoring models for mortality and rehospitalisation at a given time point and statistical significance of difference between AUC using DeLonges test (reference model GRACE score).

	Month	GRACE		CADILLAC		PAMI		TIMI		Dynamic TIMI		Zwolle	
		AUC	95% CI	AUC	95% CI	AUC	95% CI	AUC	95% CI	AUC	95% CI	AUC	95% CI
**Exitus**	*6*	0.85**	0.78; 0.93	0.82**	0.75; 0.88	0.77**^§§^	0.65; 0.80	0.72**^§^	0.70; 0.85	0.81**	0.73; 0.89	0.81**	0.73; 0.88
	*12*	0.86**	0.80; 0.93	0.82**	0.76; 0.89	0.77**^§§^	0.66; 0.80	0.73**^§^	0.70; 0.85	0.81**	0.74; 0.89	0.80**	0.72; 0.87
	*24*	0.79**	0.72; 0.86	0.76**	0.69; 0.83	0.72**^§§^	0.61; 0.76	0.68**^§^	0.64; 0.79	0.75**	0.67; 0.83	0.72**^§^	0.64; 0.80
	*36*	0.77**	0.70; 0.83	0.74**	0.67; 0.80	0.71**^§§^	0.59; 0.73	0.66**^§^	0.64; 0.78	0.73**	0.67; 0.80	0.69**^§^	0.61; 0.76
**Hospitalisation due to AHF**													
	*6*	0.76*	0.63; 0.88	0.77*	0.66; 0.88	0.84**	0.64; 0.86	0.75*	0.73; 0.94	0.83**	0.73; 0.93	0.83**	0.75; 0.92
	*12*	0.74*	0.64; 0.84	0.76**	0.67; 0.86	0.73*	0.58; 0.80	0.69*	0.61; 0.86	0.75*	0.63; 0.87	0.81**	0.73; 0.89
	*24*	0.74**	0.66; 0.83	0.80**	0.73; 0.87	0.78**	0.65; 0.84	0.74**	0.68; 0.88	0.79**	0.70; 0.89	0.80**	0.73; 0.88
	*36*	0.67*	0.58; 0.76	0.74**	0.65; 0.83	0.70**	0.58; 0.77	0.67*	0.60; 0.80	0.70**	0.61; 0.80	0.71**	0.61; 0.80

*/** Statistical significance of AUC p<0.05/p<0.001

^§^/^§§^ Statistical significance of difference between AUC using DeLonges test (reference model GRACE score) p<0.05/p<0.001

**Fig 3:**
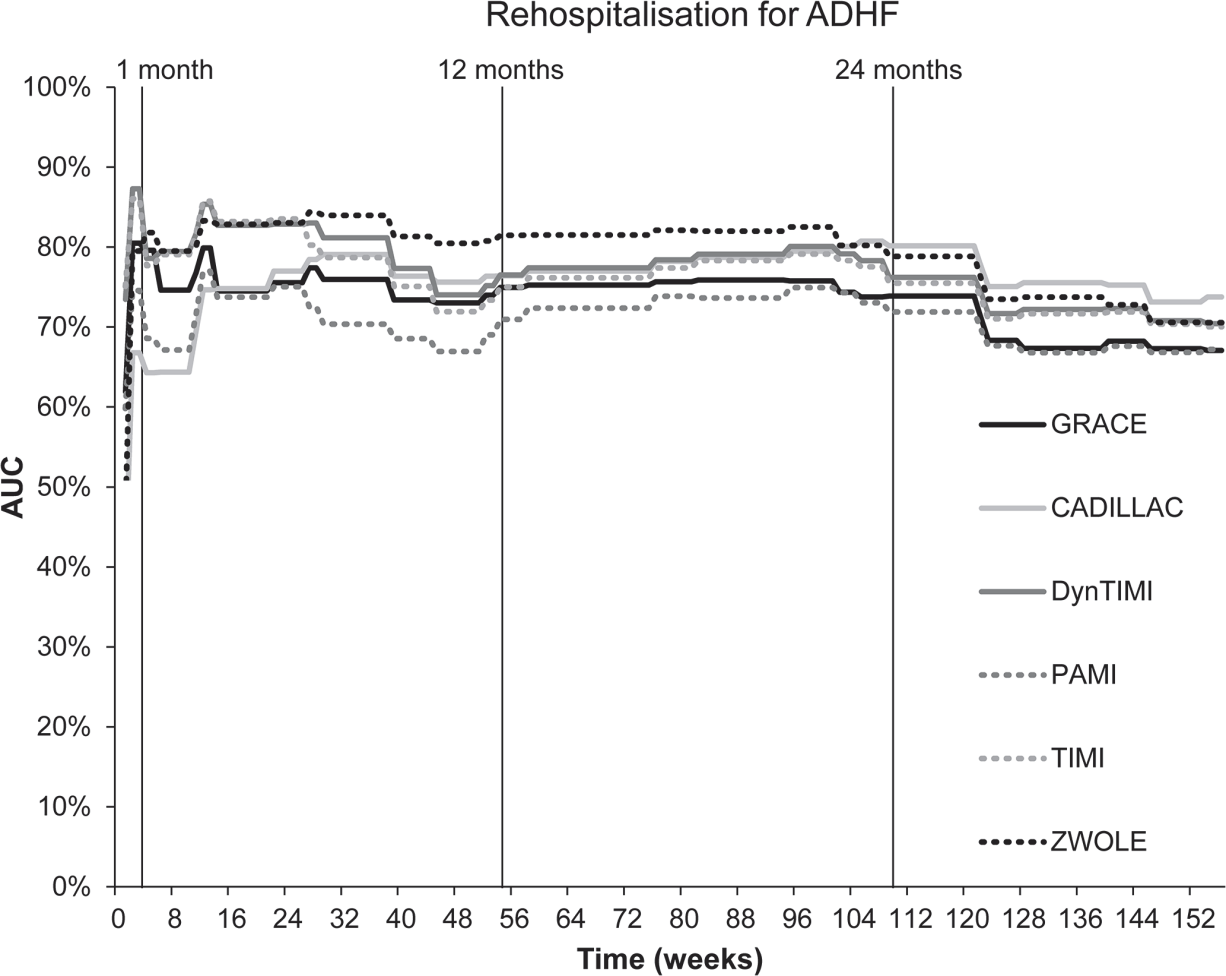
Predictive accuracy of scoring models for rehospitalisation due to ADHF during a three-year follow-up period.

The AUC of risk scores for repeat hospitalisation due to ADHF calculated by the evaluated models within the follow-up period is presented in Fig 3. PAMI had the highest predictive accuracy for repeat hospitalisation at six months, with an AUC of 0.84 (p<0.001). For long-term prediction of repeat hospitalisation, however, the precision of PAMI fell considerably; AUC decreased to 0.73 (p<0.01) at one year, 0.78 (p<0.001) at two years, and 0.70 (p<0.001) at three years (Table 5). For these time points, the Zwolle risk score was the better predictive tool: only the Zwolle score had an AUC for repeated hospitalisation at one year and two years ≥ 0.80 (one year: 0.81; two years: 0.80; both p<0.001). As for the prediction of repeat hospitalisation at three years, each risk score showed a poor predictive accuracy (0.67–0.74; all p<0.01) (Table 5). Based on the comparison of AUC values, no significant difference was found between the GRACE score (as the reference model) and other five models for the prediction of one-year risk of mortality (Table 5).

**Fig 4:**
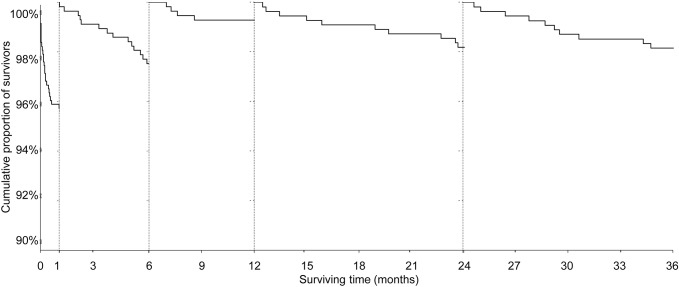
Landmark analyses of mortality after STEMI.

In order to demonstrate the influence of all variables on one-year mortality and risk of hospitalisation due to ADHF during the one-year follow-up, an univariate analysis of predictive ability was performed (Table 6). The most important predictors of one-year mortality were ejection fraction, heart failure at admission, hospital events during hospitalisation (e.g. recurrent MI, stroke, major bleed, heart failure, arrhythmia and renal failure), renal insufficiency, age, low systolic BP upon admission and cardiac arrest. TIMI flow post PCI<3, diabetes mellitus, high heart rate, 3-vessel disease and previous angina pectoris were also significant predictors of one-year mortality. Heart failure upon hospital admission, ejection fraction, hypertension, high heart rate and anterior MI or LBBB were significant predictors of one-year hospitalisation due to ADHF. The multivariate model based on all statistically significant parameters from univariate analysis was built. AUC of these models were 0.91 for 1-year mortality, and 0.84 for 1-year hospitalisation due to ADHF 0.84 (Table 6). The course of mortality by landmark analyses is shown in Fig 4. Highest mortality rates were observed in the first months of follow-up and, according to the slope of the curve, patients after STEMI could be considered to be “stable” after 6 months (Fig 4).

**Table 6:** Univariate and multivariate analysis of predictive ability for the components of given scores for prediction of 1-year mortality and one-year risk of rehospitalisation for ADHF.

	1- year mortality		Rehospitalisation for ADHF in one year	
	AUC (95% CI)	p^1^	AUC (95% CI)	p^1^
Age	0.67 (0.60; 0.75)	**<0.001**	61.3(48.9; 73.8)	0.134
Diabetes mellitus	0.64 (0.55; 0.73)	**0.002**	56.7(41.4; 72.0)	0.377
Hypertension	0.57 (0.49; 0.66)	0.118	65.5(53.5; 77.5)	**0.041**
Angina pectoris	0.60 (0.50; 0.70)	**0.027**	46.2(32.3; 60.2)	0.617
Low systolic BP	0.67 (0.76; 0.58)	**<0.001**	51.6(37.5; 65.6)	0.836
Heart rate	0.61 (0.51; 0.70)	**0.022**	66.3(50.0; 82.7)	**0.031**
Heart failure	0.73 (0.64; 0.81)	**<0.001**	73.4(60.3; 86.5)	**0.002**
Weight	0.50 (0.40; 0.60)	0.978	52.6(36.0; 69.3)	0.727
Anterior MI or LBBB	0.59 (0.50; 0.68)	0.050	65.1(51.8; 78.5)	**0.045**
Time to treatment	0.58 (0.49; 0.67)	0.080	53.7(38.9; 68.5)	0.621
Cardiac arrest	0.65 (0.55; 0.75)	**0.001**	48.4(34.1; 62.8)	0.837
Elevated cardiac enzymes	0.50 (0.41; 0.59)	0.984	50.1(35.3; 64.9)	0.991
Creatinine/renal insufficiency	0.68 (0.59; 0.77)	**<0.001**	62.3(44.0; 80.7)	0.103
TIMI flow post PCI	0.64 (0.74; 0.55)	**0.002**	48.8(33.6; 63.9)	0.871
3-vessel disease	0.61 (0.51; 0.70)	**0.019**	53.4(38.3; 68.6)	0.650
Ejection fraction	0.80 (0.87; 0.72)	**0.000**	30.3(17.1; 43.6)	**0.009**
Anaemia	0.51 (0.42; 0.60)	0.852	54.1(38.4; 69.7)	0.590
Hospital event*	0.73 (0.63; 0.82)	**<0.001**	59.3(43.1; 75.5)	0.220
Multivariate model^2^	0.91 (0.86; 0.97)	**<0.001**	0.84 (0.74; 0.93)	**<0.001**

*Recurrent MI, stroke, major bleed, CHF/shock, arrhythmia, renal failure

^1^ Statistical significance of AUC

^2^ Multivariate model consists of all statistical significant variables from univariate analysis

## Discussion

We compared six clinical prognostic scoring systems (TIMI, PAMI, CADILAC, Zwolle, dynamic TIMI, GRACE) in STEMI patients treated by pPCI according to the current guidelines by the European Society of Cardiology (ESC). These scores were developed to predict short-term survival/complications. This is the first work evaluating not only the ability to estimate the risk of one-, two-, and three-year mortality, but also the risk of hospitalisation due to heart failure.

Although the predictive accuracy expressed as the AUC for one-year mortality was slightly different, all models showed a good predictive ability. AUC values for prediction of one-year mortality obtained from our dataset by GRACE, CADILLAC and dynamic TIMI risk scores were comparable (or even better) than described originally by their authors. These models also showed a very good predictive power to estimate two- and three-year mortality. The GRACE risk score had the highest prognostic accuracy for long-term mortality at each observed time point. CADILLAC, dynamic TIMI and Zwolle risk scores had a lower accuracy but remained good predictors.

The good prognostic accuracy of the GRACE risk score was expected. The GRACE risk score is unique, as it is based on a large registry of patients across the entire spectrum of coronary syndromes, and is designed for the prognosis of all-cause mortality at six months [1,11]. The six-month prognostic value from our study (0.81) was the same as in derivation and validation sets of the GRACE registry (0.81) [1]. Kozieradska et al. [9] tested the predictive power of GRACE, TIMI, Zwolle and CADILLAC risk scores on one-month, one-year and five-year outcomes in STEMI patients. The prognostic accuracy of the GRACE risk score for one year was 0.80 in the present study. Surprisingly, in a study of STEMI patients undergoing pPCI, the GRACE risk score at one year had a particularly poor performance (0.475)[13], which is not consistent with our results. The poor accuracy of the GRACE risk score can be explained by the exclusion of patients with cardiogenic shock, cardiac arrest and the low number of AHF patients (16.4%) [13].

The CADILLAC risk score had the second-best performance in our population. At one-year follow-up, the predictive accuracy was 0.82, which is consistent with the fact that the score was developed on the basis of one-year survival analyses. In our dataset, it performed better than in the derivation and validation sets of the CADILLAC and Stent-PAMI randomised clinical trials, in which the prognostic accuracy was 0.79 and 0.78, respectively [5].

Also, in the study by Kozieradska et al. [9] the CADILLAC risk score had a lower prognostic accuracy for one-year mortality (0.74), but was comparable with the derivation and validation sets of the CADILLAC risk score. The inconsistency of our results with the results from those derivation and validation databases [5] and from the study by Kozieradska et al. [9] may be explained by the fact that high-risk-patients with cardiogenic shock were included into the analyses from those sources. However, in the above-mentioned registry of STEMI patients treated by pPCI, it performed equally well as in our analyses: the AUC at one year was 0.813 [13].

The dynamic TIMI risk score is an updated stratification model of the classic TIMI risk score, and includes hospital events. It represents a better performance of the risk stratification of STEMI patients because it monitors change over time [3]. It is designed for the prognosis of all-cause mortality at one year. In the present study, its discriminatory capacity for one-year mortality was 0.81. The TIMI risk score for STEMI also performed better in our registry than in the derivation (ExTRACT‐TIMI 25) dataset [3], in which the predictive accuracy was 0.76. The relatively small discrepancy from our results may be because the derivation dataset was a randomised trial, whereas our dataset comprised consecutive patients, and patients with cardiogenic shock were included in the analyses.

The AUC established by our research team using the Zwolle risk score was remarkably good: 0.80 for the one-year mortality. It was comparable with the results from the study by Kozieradska et al. [9], where it had a prognostic accuracy of 0.825 for one-year mortality. The model was originally developed and validated for short-term (30-day) prognoses [4] and our AUC of 0.91 for the 30-day prognosis was nearly identical to that for the derivation set of STEMI patients treated by pPCI (0.907) [4]. Therefore, the accuracy decreased significantly to 0.70 for the prediction of three-year mortality.

The AUC for the TIMI risk score for one-year mortality in our dataset (0.73) was comparable with the AUC of the model reported before. In patients from the InTIME II study, the prognostic capacity for one-year mortality was 0.725 [8]. Also, in a study of STEMI patients undergoing pPCI, the TIMI risk score had a good predictive accuracy (0.747) [13].

The PAMI model was created for predicting the six-month mortality and for the development of a very simple model in which information on the severity and time of ischaemia is excluded in comparison with the TIMI risk score. The PAMI risk score had a good predictive value for six-month mortality in the derivation sets (PAMI trials) [2], which was the same as in the present study (0.78). The predictive accuracy of PAMI was 0.77 for one-year mortality and decreased to 0.71 for three-year mortality. The AUC for one year in the present study was higher than for STEMI patients undergoing pPCI (0.75) [13]. We evaluated the mortality of patients enrolled in the period 2005–2008. Treatment modalities of patients during that time are comparable with therapy recommended by current guidelines for STEMI treatment [12]. All patients in our cohort underwent a complete functional revascularisation that was undertaken electively and guided by stress tests or fractional flow reserve [14].

Interestingly, we found that risk scoring systems established for the prediction of short-term outcomes (not longer than one-year mortality) were also useful for the estimation of three-year mortality. One explanation could be that the overall mortality after STEMI is mainly influenced by higher mortality rates during the first six months after discharge from hospital. Mortality in subsequent periods (2–3 years after STEMI) corresponds with that of patients with stable coronary artery disease. In our dataset, we excluded patients known to have cancer or other advanced diseases that could affect their prognosis and those aged >80 years. Therefore, we suppose that most deaths occurred due to cardiovascular causes.

We also showed that these scoring systems could be used to stratify patients at a high risk of hospitalisation due to ADHF, which negatively affects their quality of life and increases costs for their long-term treatment. However, the discriminatory capacity of risk scores for hospitalisation due to ADHF was not as high as that for mortality, except for the Zwolle risk score. Only the Zwolle risk score represented a higher predictive accuracy for hospitalisation up to two years than for mortality. This finding might be explained by the fact that this score includes procedural variables [4]. However, other risk scores that also include procedural variables (CADILLAC, PAMI, TIMI, dynamic TIMI) were not as good predictive tools for hospitalisation due to ADHF as the Zwolle risk score was. PAMI and dynamic TIMI risk scores had a good accuracy for the prediction of six-month hospitalisation only; for longer periods, their predictive ability decreased considerably. Our results demonstrate that heart failure upon admission, left ventricle dysfunction, adverse event during hospitalisation and renal insufficiency are the most important predictors of one-year mortality. We have developed a multivariate model based on all significant predictors; due to the limited number of patients, however, the model cannot be validated (Table 6).

Our work had four main limitations. Firstly, the study cohort was small. Nevertheless, the data put into the analyses were of high quality and patients were followed up prospectively, which ensured an adequate detection of long-term mortality and other adverse events. Secondly, further powerful mortality-prediction models for STEMI patients have been published recently. We could not test these new models in our cohort because of missing data. For example, the Syntax score (an angiography-based scoring model evaluating the severity of coronary atherosclerosis) has been validated for the prediction of one-year mortality with an AUC (c-statistic) of 0.73[15]. Furthermore, a clinical model derived from the Assessment of Pexelizumab in Acute Myocardial Infarction (APEX AMI) predicted the 90-day mortality with an AUC (c-statistic) of 0.82 and included (among others) a clinical variable denoting the total sum of ST deviation [16]. For both models, all the required data was not available. Thirdly, improvement of evaluated clinical models by adding several biomarkers was demonstrated. Although brain natriuretic peptide is a widely used biomarker and we measured its levels, we have employed more practical and bedside-applicable measurements, using only basic clinical models for our analyses. And finally, we had no case-specific data on cardiovascular mortality that could improve the predictive value of the models. Nevertheless, the all-cause mortality was also originally used as an endpoint in evaluated models.

## Conclusion

All of the tested models showed a high predictive value for the estimation of one-year mortality, but it would be more appropriate to use GRACE, CADILLAC or Dynamic TIMI risk-scoring systems for predictions for longer follow-up periods. The tested models also showed a good ability to predict the risk of hospitalisation due to ADHF, especially Zwolle, CADILLAC, TIMI and Dynamic TIMI risk scores.
